# Myocardial Recovery

**DOI:** 10.3390/diagnostics13081504

**Published:** 2023-04-21

**Authors:** Nikolaos Chrysakis, Andrew Xanthopoulos, Dimitrios Magouliotis, Randall C. Starling, Stavros G. Drakos, Filippos Triposkiadis, John Skoularigis

**Affiliations:** 1 Department of Cardiology, University Hospital of Larissa, 41110 Larissa, Greece; 2 Unit of Quality Improvement, Department of Cardiothoracic Surgery, University of Thessaly, Biopolis, 41110 Larissa, Greece; 3 Department of Cardiovascular Medicine, Heart, Vascular, and Thoracic Institute, Cleveland Clinic, Cleveland, OH 44195, USA; 4 Division of Cardiovascular Medicine, Nora Eccles Harrison Cardiovascular Research and Training Institute, University of Utah Health, Salt Lake City, UT 84132, USA; 5 School of Medicine, European University Cyprus, Nicosia 2404, Cyprus

**Keywords:** remodeling, recovery, markers, cardiac resynchronization therapy, left ventricular assist devices

## Abstract

In this paper, the feasibility of myocardial recovery is analyzed through a literature review. First, the phenomena of remodeling and reverse remodeling are analyzed, approached through the physics of elastic bodies, and the terms myocardial depression and myocardial recovery are defined. Continuing, potential biochemical, molecular, and imaging markers of myocardial recovery are reviewed. Then, the work focuses on therapeutic techniques that can facilitate the reverse remodeling of the myocardium. Left ventricular assist device (LVAD) systems are one of the main ways to promote cardiac recovery. The changes that take place in cardiac hypertrophy, extracellular matrix, cell populations and their structural elements, β-receptors, energetics, and several biological processes, are reviewed. The attempt to wean the patients who experienced cardiac recovery from cardiac assist device systems is also discussed. The characteristics of the patients who will benefit from LVAD are presented and the heterogeneity of the studies performed in terms of patient populations included, diagnostic tests performed, and their results are addressed. The experience with cardiac resynchronization therapy (CRT) as another way to promote reverse remodeling is also reviewed. Myocardial recovery is a phenomenon that presents with a continuous spectrum of phenotypes. There is a need for algorithms to screen suitable patients who may benefit and identify specific ways to enhance this phenomenon in order to help combat the heart failure epidemic.

## 1. Introduction

The heart shows changes in its structure and morphology. This occurs either as a physiological response of the organism when subjected to a physiological stimulus (physical growth, exercise, etc.) or as a pathological response to harmful factors that cause volume, pressure overload or direct cellular damage in combination with the subsequent excessive activation of the renin-angiotensin axis and the sympathetic system. In several cases, either after the remission of the aforementioned harmful factors or the application of an appropriate therapeutic approach, there is a reversal of both the macroscopic anatomical abnormalities and the restoration of cellular structures and functions with a tendency to return to the pattern of the normal heart. In this way, the heart manages to improve its cardiac performance to pre-damage levels despite the hemodynamic disturbances that may continue to exist. This phenomenon is called reverse remodeling. On the occasion of the above data collection, the researchers created a term they called myocardial recovery. According to this definition, myocardial recovery is defined as the morphological and functional recovery of the heart either automatically or after one or more interventions while meeting the following criteria: (1) absence of recurrence of heart failure events and (2) freedom from future heart failure events [1]. In the international bibliography, this specific phenomenon was intensively studied in the context of the question of whether reverse remodeling is identified with the concept of myocardial recovery. In the first criterium, the cardiac tissue recovers morphologically and functionally, but without ensuring that the second criterium above is satisfied. Thus, it follows that the two concepts are not identical as reverse remodeling is a necessary condition for achieving myocardial recovery, but it is not sufficient without the fulfillment of the two aforementioned criteria. Therefore, myocardial recovery of the heart requires macroscopic and microscopic morphological and functional restoration of the myocardium to the extent that it brings about present and future freedom of the patient from heart failure and its complications.

In this paper, through a systematic review of the international literature, the feasibility of myocardial recovery is studied, the mechanisms that contribute to its achievement, possible molecular markers for its identification, interventions that are capable of bringing about the above result based on the modification of specific pathophysiological mechanisms and, finally, the recognition of specific characteristics of patients who may experience myocardial recovery and the criteria for their detection [1,2].

## 2. Pathophysiology

Remodeling of the heart causes changes both macroscopically, involving its mass and geometry, and microscopically, involving two factors. The first is the cardiomyocyte in which the following changes take place: (1) cellular hypertrophy with modification of the structure, constitution and histophysiology of myofibrils as well as changes in their alignment in space (in series, parallel), (2) remodeling of the protein coupling and communication structures among them, (3) changes in the physiology of cell groups at the level of metabolism, signaling, apoptosis as well as the effect of the neurohormonal axis on the above and (4) the modification of the genome at all levels of gene expression. The second is the extracellular basic substance with changes that take place in its structure, composition and quantity. Several possible mechanisms have been proposed to achieve reverse remodeling. The predominant approach to the physiology of this phenomenon is related to the deformability of myocardial fibers. We know from mechanics that, in a material, when an increasing tension is applied to it, it can increase its length up to a certain point where, if the applied tension is interrupted, it can return to its original state without affecting its structure (elastic deformation). From this point onwards, the material will partially return to its original state as permanent structural changes are created in its structure (plastic deformation). Myocardial tissue shows a similar behavior. When exerting tension on the myocardial wall either due to increased volume or pressure, the damage it will suffer can be either permanent or reversible in whole or in part. In the second case, after the removal of the damaging factor, the heart gradually tends to restore its geometry and its thickness to a certain extent, depending on the type and severity of the damage, the length of time it suffered and the effect of additional factors such as the degree of activation of the neurohormonal axis.

**Figure 1:**
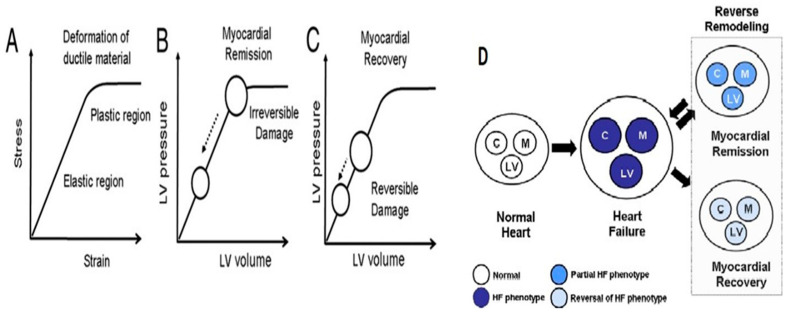
(**A**) A material, when an increasing stress is applied to it, can increase its length up to a certain point and, when the applied stress is stopped, it can return to its original state without affecting its structure (elastic deformation). From this point onwards, the material will partially return to its original state as permanent structural changes are created in its structure (plastic deformation). (**B**,**C**) Myocardial tissue shows a similar behavior. When tension is exerted on the myocardial wall, the damage it will suffer can be either permanent or reversible, in whole or in part, depending on the damage and its duration. (**D**) The three factors that will determine the evolution of myocardial functionality are (1) its macroscopic geometry, (2) the cardiomyocyte and (3) the extracellular matrix. The clinical impact that the degree of remodeling will have concerns two possible outcomes, myocardial remission and myocardial recovery. Abbreviations: C, cardiac myocyte; M, extracellular matrix; LV, left ventricle. Reprinted with permission from Mann DL, et al., (2012), Copyright © 2012, American College of Cardiology Foundation. Published by Elsevier Inc. Ref. [1].

Therefore, reverse remodeling is directly related to the extent of damage the myocardium undergoes at the microscopic level from the effect of the plastic deformation it has undergone. The three factors that will determine the evolution of the functionality of the myocardium are (1) the macroscopic geometry of the heart as the structure of its cavities directly affects its hemodynamic function, (2) the cardiomyocyte and (3) the extracellular matrix. Depending on the degree of damage and the dysfunctions that the above will show, the degree of reverse remodeling of the heart will be determined. In turn, the clinical impact that will be brought about by the degree of remodeling, and specifically the absence of recurrence of heart failure due to the improvement of the structure and function of the myocardial tissue and also the freedom from future heart failure events due to the residual damage that will remain in the tissues, will also determine the clinical outcome. The two possible outcomes are myocardial remission where we have reverse remodeling without meeting the above two criteria and myocardial recovery (Figure 1) where, in addition to the macroscopic recovery of the myocardium, the corresponding clinical benefit to the patient is presented as defined [1].

## 3. Biomarkers

The scientific community has put a lot of effort into defining various indicators such as biomarkers, molecular, imaging techniques, etc. to identify reverse remodeling and by extension myocardial recovery with the aim of rapid and early stage response to potential therapies. In this particular paper, the possible indicators that have been mentioned in the international literature and the possibilities they provide in the clinical and research fields are reviewed.

### 3.1. Biochemists

#### 3.1.1. Natriuretic Peptides (Brain Natriuretic Peptide (BNP), N-Terminal Pro–B-Type Natriuretic Peptide (NT-proBNP))

From the PROTECT (ProBNP Outpatient Tailored Chronic Heart Failure) study, a positive correlation of natriuretic peptides with left ventricular end diastolic volume (LVEDV), left ventricular end systolic volume (LVESV), right ventricular systolic pressure (RVSP) and inverse with left ventricular ejection fraction (LVEF), E/e’ and right ventricular function was observed [3]. Further, from a study of patients with cardiomyopathy of pregnancy it was observed that, when NT-proBNP ≥ 900 pg/mL, there is a lower probability of recovery of LVEF and LVEDV [4]. Finally, regarding ischemic disease, increased levels of this in non-ST elevation myocardial infarction (NSTEMI) patients were associated with improved left ventricular (LV) function over 8 months [5] and decreased levels of this with improved right ventricular (RV) function in patients with ST elevation myocardial infarction (STEMI) [6]. Consequently, natriuretic peptides do not directly predict myocardial recovery but are associated with morphological and functional changes in response to treatments, indirectly indicating the potential for reverse remodeling.

#### 3.1.2. Troponin

In a secondary analysis of the ProBNP Outpatient Tailored Chronic Heart Failure (PROTECT) study, elevated troponin values were associated with an increased likelihood of LV remodeling in chronic heart failure (HF) [7]. At the same time, from the Prediction of ICD Treatment Study (PREDICTS) study, its increased values in HFrEF after an acute coronary event were found to be an independent predictor of systolic dysfunction [3], while its reduced levels in patients with STEMI were related to improved RV function [6]. From the above, it also follows that it is not a direct indicator of myocardial recovery but an indicator of remaining ischemic damage and, indirectly, of a reduced possibility of reverse remodeling.

#### 3.1.3. Soluble ST2 (sST2)

It is a transmembrane receptor in cardiomyocytes, fibroblasts and endothelial cells where it is produced in increased myocardial stress and binds IL-33, playing a serious role in fibrosis and in general in the course of heart failure. In studies, its increased levels were associated with increased left ventricular dimensions and volumes, reduced ejection fraction and worse right ventricular function. Conversely, levels < 35 ng/mL were associated with reverse remodeling.

#### 3.1.4. Interleukin-8 (IL-8)

Studies in patients with STEMI, angioplasty and those hospitalized with heart failure as a complication showed a reduced likelihood of improvement in left ventricular contractility when elevated serum IL-8 levels were observed [8].

### 3.2. Electrocardiogram

From the electrocardiogram (ECG) we can draw indirect conclusions about the possibility of improving myocardial function. Persistent ST elevation after successful luminal opening in patients with STEMI is considered a sign of poor microvascular circulation and a negative predictor of increased ejection fraction at 3 months [9]. Further, repolarization disturbances can add additional information. In a study of patients with non-ischemic heart disease with an ejection fraction <40% and a first hospitalization for heart failure who improved their ejection fraction, a shorter angle between the QRS-T complexes, a shorter QTc interval and a less negative distribution of the JT interval area were observed. In addition, the first was related to the longitudinal and circumferential deformation of the myocardium and the third to an increased ejection fraction, reduced dimensions of the left ventricle and to longitudinal deformation [10].

### 3.3. Visual Techniques

Of the existing imaging techniques from this literature review, it appears that (1) the fractional flow reserve (FFR) can identify viable post-infarct myocardium that has an increased chance of recovery after angioplasty [11], (2) non-invasive coronary flow reserve (CFR) less than 24 h from myocardial infarction is an independent predictor of left ventricular function recovery [12] and (3) the index of microcirculatory resistance (IMR) in thrombolytic STEMI patients with subsequent angioplasty is associated with left ventricular function recovery [13].

### 3.4. Molecular Techniques

#### 3.4.1. MicroRNAs

MicroRNAs are small single-chain nucleotide molecules that regulate the inhibition of mRNA and, by extension, the regulation of gene expression. From studies, the most important of these are the following:

- miR 23a, 195, 21, 24, 125b, 195, 199a: associated with myocardial recovery after LVAD support patients;
- miR-26b-5p, miR-145-5p, miR-92a-3p, miR-30e-5p and miR29a-3p: found elevated in cardiac resynchronization therapy (CRT) patients who responded to treatment;
- miR-30d: increased in responders to CRT placement (strong association) and also associated with an improvement in LVEF [14].

#### 3.4.2. Other Indicators

- IGF-I mRNA: from studies, it was observed to be increased in patients who received a left ventricular assist device (LVAD) with the drug protocol of Harefield and who showed myocardial recovery [15];
- Fas and TNFR1: these two molecules were studied in the Intervention in Myocarditis and Acute Cardiomyopathy (IMAC) study and their increased values were negatively related to the improvement of myocardial function in recent-onset cardiomyopathies, possibly reflecting an increased apoptotic process [16].

From the above data, it follows that there is no molecular, imaging or biochemical marker capable of predicting myocardial recovery as such. Their use lies more (depending on the heart disease) in the identification of the myocardium, which shows an improvement in its functionality which is essentially an indirect element of reverse remodeling. Therefore, they can be used as auxiliary elements for the screening of patients who, with appropriate clinical consideration, will show an increased probability of myocardial recovery.

## 4. LVAD Therapy

The use of LVADs for left ventricular unloading is a cornerstone in the treatment of advanced heart failure. Historically, in the 1990s, the FDA approved their placement in patients to stabilize them until a compatible graft was found for transplantation. After observations that the device was well tolerated by patients, its use was extended as a palliative treatment measure. In addition, an observational study of patients with cardiogenic shock in the context of dilated cardiomyopathy resulted in cardiac recovery in some of them, with the possibility of their being weaned from the device [17]. Then, in 2001, the Randomized Evaluation of Mechanical Assistance for the Treatment of Congestive Heart Failure (REMATCH) study was published, comparing drug therapy with the additional use of an LVAD. The results showed an increase in 1-year survival, an improvement in quality of life and in the functional New York Heart Association (NYHA) classification in the second group [18]. Since then, intensive research has been carried out internationally into the possibility of myocardial recovery through this technique. Possible pathophysiological mechanisms that are modified after artificial left ventricular unloading are analyzed below.

### 4.1. Extracellular Matrix

**Figure 2:**
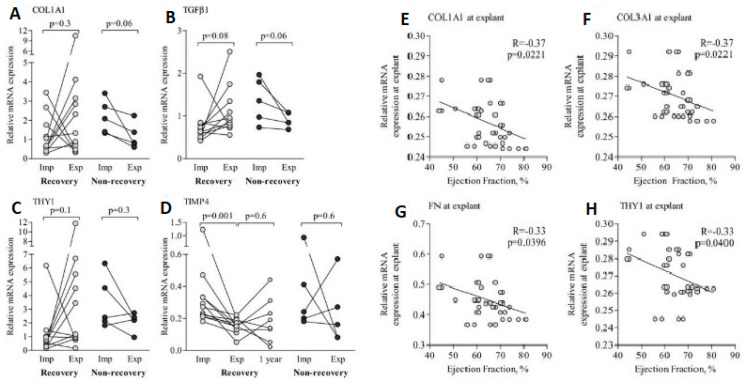
(**A**,**B**): The changes in the mRNAs of COLIAN1, TGF1β, in which no statistical difference is observed before and after the removal of the LVAD. (**C**,**D**): The changes in the mRNAs of THY1 and TIMP4, where there is a statistically significant difference between the patients who recovered and those who did not. (**E**–**H**): mRNA expression of COL1A1, COL3A1, FN and THY1 after LVAD removal was negatively correlated with ejection fraction. Reprinted with permission from Felkin LE, et al., (2009), Copyright © 2009 International Society for Heart and Lung Transplantation. Published by Elsevier Inc. Ref. [19].

In heart failure, remodeling of the extracellular matrix is observed, causing fibrosis and abnormal degradation of its components. The consequence of this is the dysfunction of many cellular functions, contributing to the disruption of systolic and diastolic function. With the use of LVAD, changes have been observed both in the composition of the extracellular matrix and in the expression of the genes related to it. A related study was conducted at Harefield Hospital, where patients with dilated heart disease of non-ischemic etiology were selected and given an LVAD due to worsening cardiac function under a specific medication protocol. Ventricular biopsies were then obtained before LVAD implantation, at removal from recovered patients and at one year after device removal. This was followed by analysis of a series of genes and proteins that play a key role in the fibrosis of the extracellular matrix. Comparing the patients who ascended to those who did not ascend, the following conclusions are drawn: (1) the pro-fibrotic markers COL1A1, TGF-β1, THY1 were increased before LVAD implantation in the patients who did not ascend compared to those who ascended, (2) between the two groups no significant differences in gene expression levels were observed after treatment, (3) the pro-fibrotic genes COL1A1, COL3A1, FN and THY1 had a negative correlation with the ejection fraction in the patients who recovered and (4) when analyzing the expression of each gene separately, a decrease in the expression of the TIMP4 gene was observed in the patients who showed recovery, while in the rest there was a separate pattern of increase and decrease for each patient (Figure 2) [19]. The TIMP4 gene has also been observed to decrease the protein limitin after LVAD placement, which is detected at increased levels in heart failure. In addition, there is evidence in other studies of an increase in the amount and functionality of metalloproteases (MMPs) in heart failure [20] leading to increased matrix degradation and collagen deposition associated with fibrosis, with the ultimate result of distension and dysfunction of the heart. After the use of LVAD, a decrease in MMP-1, MMP-9, an increase in the metalloprotease inhibitors TIMP-2, TIMP-4 was observed while the ratio of mutated collagen to total increased, the latter being an indication of repair of the extracellular matrix [21,22]. Therefore, the use of LVAD may facilitate the restoration of the physiological structure of the basic substance, an element necessary for the homeostasis of cardiomyocytes. Finally, the analysis of the markers COL1A1, TGF-β1, THY1 and the genes COL1A1, COL3A1, FN and THY1 could in the future be markers of recovery and guide decision making for the removal of mechanical circulatory support systems.

### 4.2. Protein Changes

In addition to extracellular matrix proteins, a number of other structural and functional proteins have been observed to be altered in quantity and functionality after LVAD placement. An example is the creatine kinase (CK), which has been associated with the energetic modifications that the heart undergoes in heart failure. Biochemically, CK subunits can be composed of 3 isoforms, CK-B, CK-M, CK-Mt. The first is found mainly in fetal heart tissue and in a minimal amount in adults, while the third is found in the inner mitochondrial membrane. In a study of heart failure, a decrease in the activity of total CK, CK-M, CK-Mt isoforms and an increase in CK-B associated with an increase in wall stress have been observed. After LVAD placement, an increase in total CK activity, an increase in protein expression of CK-M, CK-Mt isoforms to the extent of normal cardiac function and no change in CK-B were observed [22]. These changes after mechanical support of the heart are an indication of the importance of the role of mechanical support improving the activity of the CK enzyme involved in the energetic and overall recovery of the heart. After the use of LVAD, a decrease in p44/42, JAK ½ and an increase in p38 were observed, with a parallel histological decrease in cell hypertrophy and apoptosis [22]. Finally, at the level of protein translation, a reversal of the amino-terminal end of dystrophin (a protein that plays an important role in the occurrence of heart failure) and a decrease in the phosphorylation of Troponin I have been observed after LVAD placement [20].

### 4.3. Apoptosis

Apoptosis is one of the most important processes in the pathogenesis of heart failure. From studies comparing heart tissues from patients with ischemic and non-ischemic dilated heart disease compared to normal heart tissues, leakage of cytochrome c from the mitochondria into the cytoplasm was observed in the former, while, in the control group, it remained in the mitochondria. The ability of cytochrome c to activate the apoptosis mechanism when transported to the cytoplasm is known. Studying the mechanism of apoptosis in heart failure results in a decrease in the activation of the Flip-L and S proteins (caspase 8 antagonists), resulting in the activation of caspase 8, which causes the truncation of the Bid protein (inducer of the release of cytochrome c caused by caspase 8) which leads to the release of cytochrome c from the mitochondria and the activation of caspase 3. Cells, in an attempt to reduce the effects caused by overactivation of apoptosis in heart failure, reduce the activation of XIAP (inhibitor of active caspase 3), of Smac-L (a second activator of caspases originating from the mitochondria) and increase the activation of Smac-S, leading to a limitation of caspase 3 activity while at the same time a reduction of DNAases is also observed in the cells. Additionally, apoptosis, as a fairly energy-consuming process, can cause damage to the enzymes of the respiratory chain and oxidative phosphorylation due to overactivation. All the above processes aimed at reducing the fragmentation of the nucleus and reversing apoptosis lead to the preservation of the integrity of the nucleus but, at the same time, the breakdown of contractile proteins of the cell, causing cellular and subsequently cardiac systolic dysfunction.

Because of the preservation of the nucleus, the possibility of reversal of cellular damage after circulatory support with LVAD was investigated. Studies conducted have studied samples of heart tissue from patients before LVAD placement and at the time of their removal when those patients were transplanted and also from donor heart tissues. An increase in the activation of apoptosis reversal genes and proteins such as FasEx06 Del and Bcl-xL and an almost freeing of cytochrome c from the sarcoplasm without a complete restoration of its amount in the mitochondria were observed. Therefore, the reduction of cytochrome c from the sarcoplasm can be an indicator of the degree of reversal of apoptosis and has been associated with the process of reverse remodeling. Thus, it can predict and explain the existence of “responders” with the use of LVAD systems essentially based on the correlation of the amount of cytochrome c and the degree of apoptosis. In this way, it predicts the possibility of cell recovery from this reversal [22]. Finally, studies after LVAD placement have also observed a reversal of the decrease in the amount and functionality of the 20 s proteasome, which is implicated in the deposition of undegraded proteins in cardiac tissue, a decrease in autophagy marker Atg5-Atg12 complexes and LC3 which is increased in cardiac hypertrophy [2] and a decreased altered expression of proliferative repair markers (proliferating cell nuclear antigens 5, 6, 10, 37) [23].

### 4.4. Sarcoplasmic Reticulum

Very important conclusions emerge from the study of the relationship of the sarcoplasmic reticulum, the sarcolemma and the cytoskeleton with cardiac recovery and the use of LVAD. In heart failure, there is an increase in the proteins β-tubulin (with hyperpolymerization), talin, spectrin (resulting in an increase in the stiffness of the heart muscle) and a decrease in the proteins titin and α-myosin of the heavy chains, causing a decrease in the elasticity of the myocardium. In a study at Harefield Hospital in patients with dilated cardiomyopathy, biopsies were performed at LVAD implantation and at removal after observed clinical cardiac recovery. Histochemistry analysis showed: (1) an increase in cytoskeletal actin, αII spectrin, α-smooth muscle fiber actin and vimentin, (2) a decrease in talin, β-tubulin and vinculin proteins, (3) a general tendency to increase sarcomeric proteins due to LVAD placement and at the same time a reduction of adhesion proteins with the exception of actin and (4) specific proteins such as vimentin, αII spectrin, β-tubulin are expressed not only in cardiomyocytes but also in different cell populations and possibly in stem cells that promote recovery. Regarding the gene changes in molecular control of patients with LVAD placement who did not have their heart resuspended, an increase in the gene expression of 17 genes responsible for sarcomeric proteins was observed after LVAD placement, all of which were expressed at the protein level [24]. Additionally, it is noteworthy that, after LVAD placement, there is increased activation of the Laminin A/C genes which modify the proteins of the intermediate filaments of the sarcomere and β-integrin, where they favor communication between the cytoskeleton and extracellular matrix and are involved in many physiological and pathophysiological pathways [25].

Another element that emerges from the microscopic study of cell anatomy is the fact of T-tubule remodeling in heart failure and the reversal of remodeling upon LVAD placement. It has been shown that the degree of T-tubule remodeling affects the ability of the myocardium to recover. The more severe the degree of remodeling, the less likely it is to revive, especially when the tubules have a “sheet” shape. Studies have shown that this type of remodeling does not lead to recovery after LVAD placement. The most likely explanation comes from an electron microscopy study, where an increase in the distance between ryanodine receptors (RyR) and the L-type calcium channels of the tubule membrane was observed, disrupting the coupling of excitation and contraction of the cell [26].

**Figure 3:**
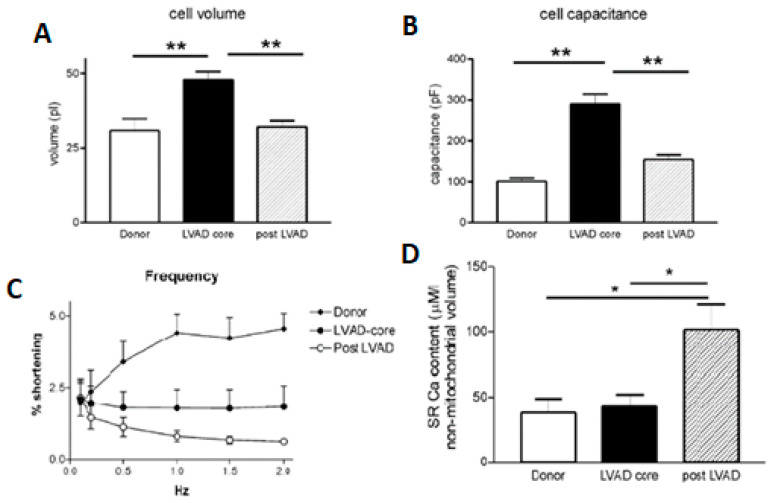
After LVAD placement (**A**): cell size and (**B**): capacitance (an index of cell surface area), tend to return to normal donor values, indicating reversal of hypertrophy. (**C**): No increase in the contraction-frequency-dependent relationship was observed in patient cells with or after LVAD use in contrast to donor cells. As a result, the researchers hypothesized the involvement of calcium transport mechanisms as responsible for the improvement in contraction. (**D**): LVAD-implanted HF patients show reduced calcium current amplitude compared to LVAD-removal patients and no-difference compared to donors. After LVAD removal there is an increase in the amount of calcium observed compared to heart failure patients and donors. The LVAD increases the amplitude of the calcium current and its quantity, improving the excitation-contraction relationship of the cardiomyocyte, being a mechanism for promoting cardiac recovery. * *p* < 0.05; ** *p* < 0.01; LVAD core: left ventricular tissue taken at LVAD implant; post LVAD: left ventricular tissue taken at LVAD removal. Reprinted with permission from Terracciano CMN, et al., (2003), Copyright © 2003, Oxford University Press [27].

In addition to the study of the remodeling of the structure of the sarcoplasmic network, studies were also carried out to highlight its pathophysiological changes. In particular, a study was carried out at Harefield Hospital with cardiac histological samples taken from patients before LVAD placement and after or during its removal due to clinical improvement or transplantation [27]. Additionally, donor hearts were sampled and all patients were under a specific medication protocol. A return of cells to their normal size was observed after LVAD removal compared to twice that observed before implantation of the systems, as well as a decrease in cell capacity and an increase in the capacity-to-volume ratio, findings consistent with a regression of cell hypertrophy. Consistently, a longer time to contractility and decay was observed in myocardial cells from LVAD patients compared to those of the other groups that were the same, while no increase in the contractility-frequency-dependent relationship was observed in cells from patients with or after LVAD use in contrast to donor cells. As a result, the researchers hypothesized the involvement of calcium transport mechanisms as responsible for the improvement in contraction. To prove the above hypothesis, the relationship of calcium current density between groups was studied at various voltages, showing that LVAD patients showed a reduced calcium current amplitude compared to those who underwent LVAD removal and donors where no difference was observed. In this way, based on previous research data, the role played by excitation–contraction coupling is indicated, which is reduced in HF and recovers after completion with LVAD therapy. In addition, the change in the total amount of calcium in the sarcoplasmic reticulum was studied, resulting in a non-significant difference between subjects with LVADs and donors, but a significant increase in patients after successful removal of the device (Figure 3).

Summarizing the study for the use of LVAD systems and sarcoplasmic reticulum results: (1) reduction in cell size through reduction in cell capacity and volume as an indication of reversal of hypertrophy but not returning to control values, (2) faster contractility and decay, indicating possible clinical improvement through normalization of calcium current amplitude and amount and (3) the force–frequency relationship showed no improvement (possible non-cardiac recovery factor) [27]. Therefore, we can conclude that LVAD placement brings about significant changes in the structure, morphology and functioning of the sarcoplasmic network that may be a mechanism of clinical recovery in treatment with long-term circulatory support.

### 4.5. B-Receptors

The involvement of β-adrenergic receptors in the pathophysiology of heart failure has been demonstrated and is the subject of intensive study. Examining them structurally and functionally, these receptors are called GPCRs (G protein-coupled receptors) and are divided into 4 subtypes β1–β4. When the catecholamines bind to the receptors, the G protein is activated, which is connected to them and which consists of 3 subunits α, β, γ. Upon its activation, it exchanges the GDP bound to it for GTP and causes uncoupling of the now active α and β subunits, activating corresponding adenylate cyclases or phospholipases for further molecular signaling. At the β1 receptor, the α subunit activates adenylate cyclase which produces cAMP and this activates PKA. The last phosphorylates troponin I, calcium channels and phospholamban increasing myocardial contractility [28].

In heart failure, the increased activation of the neurohormonal axis leads to the release of large amounts of catecholamines. This leads to increased activation of the receptor and G protein where the latter activates GRK2 (G protein receptor kinase-2) or βARK1. GRK2 phosphorylates β1, β2 receptors, to which arrestin protein will then bind, causes functional uncoupling between the G protein and receptor. The result is the deactivation of the receptors, a process called desensitization. This leads to the downregulation of 50% of the β-1 receptors and the desensitization of the rest, with the ultimate result being the impairment of contractility. The above mechanism is supported by studies in which, in heart failure, a reduced activity of adenylate cyclase is observed before and after activation by isoproterenol compared to healthy tissue samples, while stimulation of the α-subunit of the G protein by sodium fluoride causes the same degree of cyclase activation in subjects with heart failure and subjects in the control group. Thus, the damage as a dominant version is located between the receptor and G protein and the importance of GRK2 in heart failure is indicated, explaining the increased activity observed in it. In addition, increased activity of Gαi (G protein inhibitor of adenylate cyclase) and a decrease in β-receptor density were observed in heart failure compared to the control group.

After LVAD placement, a decrease in GRK2 activity, expression and mRNA levels, an increase in adenylate cyclase activity, a decrease in Gαi expression and an increase in β-adrenergic receptor density were observed. Therefore, we can conclude that the mechanical unloading of the left ventricle brings about an improvement in the function of β-receptors. In fact, experimental data indicate a non-statistical difference between samples from LVAD patients and samples from the control group [29]. In addition, changes in cardiac GRK2 were correlated with changes in GRK2 in blood lymphocytes, being a possible future biomarker, something that is also confirmed in animal models [30].

Presumably, the device, through hemodynamic changes, brings about modifications in the neurohormonal environment locally. These specific changes may be the driving force for the improvement to levels resembling restoration of adrenergic signaling in the cardiomyocyte. The pivotal role of GRK2 in the pathophysiology of heart failure and the diagnostic possibilities it may offer in the future are also highlighted. The final result of the above changes is the improvement of the contractility of the heart muscle and, by extension, the systolic function.

### 4.6. Energy

An important role is also played by the energetic changes that the heart undergoes both in heart failure and after LVAD placement. A study conducted looked at specific chemicals and genes that regulate key points of cardiomyocyte metabolism. For this reason, histological samples were taken from patients with heart failure before and after LVAD use and from hearts with normal left ventricles. At the gene level, the variations in the expression of specific transcription factors as well as mitochondrial genes necessary for metabolism were studied. It has been observed that, in heart failure, we have a decrease in the transcript levels of peroxisome proliferator-activated receptor coactivator 1 (PGC1A) and estrogen-related receptors (ERRA, ERRG), which are important metabolic control elements. A decrease was also observed in the expression levels of genes involved in fatty acid metabolism such as carnitine palmitoyltransferase 2 (CPT2), very long chain acyl-CoA dehydrogenase (ACADVL) and 3-hydroxyacyl-coenzyme A dehydrogenase (HADHA). An additional decrease was also observed in the transcription of genes involved in pyruvate metabolism and therefore in the regulation of the Krebs cycle such as pyruvate monocarboxylase cotransporter (MCT1), pyruvate dehydrogenase (PDHB), malic enzyme (ME3) and pyruvate/alanine aminotransferases (GPT). In glucose management, the results indicated a decrease in glucose cotransporters (GLUT1, GLUT4) and phosphofructokinase (PFK). Finally, a decrease was also observed in the expression of the genes that regulate the functioning of the respiratory chain and of mitochondrial transcription factor A (TFAM), which is involved in the replication of mitochondrial DNA.

Then, using special biochemical techniques, the fluctuations of specific chemical substances involved in the production of energy in the heart cell were also studied. Specifically, there was a decrease in short and medium chain acylcarnitines from C2–C10 and especially acetylcarnitine (C2) (acetyl-CoA substitute), propionylcarnitine (C3), isovalerylcarnitine (C5), succinylcarnitine (C4-DC), butyrylcarnitine (C4) and little difference in concentrations of longer chain acylcarnitines (C14 and above). The above, depending on the biochemical processes involved, may be glucose, fatty acid or amino acid oxidation products. Further, pyruvate levels were increased, Krebs cycle intermediates decreased and α-ketoglutarate levels remained stable. Finally, amino acids that are a source of alternative substrates for the Krebs cycle, such as alanine, glutamine/glutamic acid, leucine/isoleucine and citrulline, were also found to be reduced in heart failure. Continuing the study of the above substances, a positive correlation emerged between ejection fraction and short-chain, medium-chain acylcarnitines and malic acid, and a negative correlation with pyruvate. There was also a positive correlation between LVEDd and pyruvate and a negative correlation with short- and medium-chain carnitines as well as with several Krebs cycle intermediates.

The above observations, and especially the correlations of the echocardiographic indices with specific metabolic products, the increase of pyruvate and the decrease of intermediate factors of the Krebs cycle, are important findings for clarifying the role of energy remodeling in heart failure. The above findings also support the modification in the expression of genes with a pivotal role in important metabolic processes. In addition, the decrease in the regeneration of mitochondria and the decrease in the expression of the mitochondrial genes responsible for metabolic activities are another element of the alteration of the transcriptional regulation of the myocardial energy production processes. Through all these changes, the myocardial cell is driven to suppression of the pyruvate metabolism. This, combined with the reduction of fatty acid oxidation, the reduction of the amount of acetylcarnitines (an indirect indicator of the amount of oxidation substrates), the reduction of the amount of amino acids (alternative sources of oxidative substrates) as well as the involvement of pyruvate in the oxidation of glucose and fats of acids, lead to a generalized poverty in the amount of oxidizing substrates. This in turn results in hypofunction of oxidative phosphorylation resulting in a deficit in ATP production in the cardiomyocyte. This energy poverty disrupts the cell’s contractile capacity, making it vulnerable to stress at the same time as it shows reduced energy reserves for adaptation. Experimental inactivation of the above genes in animal models caused damage to their myocardium, confirming the correlation of the change in the expression of the specific genes in the presence of heart failure.

In contrast to the use of LVAD, restoration of the levels of acylcarnitines with C2 to C10 chains, pyruvate, amino acids and phosphofructokinase activity was observed as well as restoration of several metabolic pathways. Further, at the gene level, an improvement was observed in the main transcription factors and co-regulators and especially PGC1A, PGC1B and ERRG, an increase in the activity of the mitochondrial genome and in particular the genes responsible for metabolism, mitochondrial biogenesis, the respiratory chain and oxidative phosphorylation as well as an increase in the activation of genes related to fatty acid and pyruvate metabolism. In addition, in heart failure, there is an increase in p53 protein due to a decrease in the length of telomeres of specific genes which causes a decrease in the expression of the transcription factors PGC1A and PGC1B. This, as a result, leads to the suppression of the oxidation of fatty acids and pyruvate. With LVAD placement, a decrease in p53 protein expression and restoration of the above metabolic pathways, as well as the telomere gene length of the cardiomyocyte, were observed [31].

Another notable feature observed in heart failure is the alteration of the metabolism by reducing the oxidation of fatty acids, which is the preferred energy source, and diverting it to increase glycolysis, leading to a fetal pattern of energy management. Nevertheless, in the above studies there is no correspondence between the increase in glucose with an increase in the intermediate products of the Krebs cycle, even after the use of LVAD, resulting in a mismatch between glycolysis and oxidative phosphorylation. In the literature, two etiologies are considered as possible. The first considers the unloading of the ventricle after LVAD placement as a factor of significant reduction of the required energy of the myocardium, and for this reason the production of ATP only from the process of glycolysis is sufficient for the energy needs of the heart. The second examines the channeling of glucose into alternative metabolic pathways. The two most prevalent are the pentose phosphate metabolism and the one-carbon metabolism. In the first we have energy production, nicotinamide adenine dinucleotide phosphate (NADPH) production and production of specific pentose sugars which are converted into ribitol. Ribitol is an essential element for the glycosylation of α-dystroglycan (α-DAG1), which plays an important role in the communication between the extracellular matrix and cells in order to maintain the structure and function of muscles. The second metabolic pathway leads to the production of purines and NADPH without channeling glucose to the hexosamine and polyol metabolic pathways involved in the pathophysiology of heart failure. Therefore, by diverting glucose metabolism from oxidative phosphorylation, the cell increases the production of the structural components necessary for its repair, NADPH that protects against free radicals that promote oxidative stress and also promotes the production of regulatory molecules of various important functions of the cell.

The above evidence is supported by studies in which LVAD placement was studied with the metabolic changes experienced by responders compared to non-responders and normal donors. The following emerged from the study: (1) a decrease in the levels of nucleotides in responders compared to non-responders where they were increased and an additional increase in the amount of ribosomes in the former, indicating an increase in the transcriptional and translational process, (2) decreased levels of NADPH and an increased ratio of NADP/ NADPH before and after LVAD placement in non-responders and vice versa in responders, (3) increased 4-HNE levels associated with increased oxidative stress in non-responders and decreased oxidative stress in responders and (4) decreased mitochondrial volume density and distribution of mitochondria in non-responders in contrast to responders, where there was an increase in mitochondrial volume density and distribution of mitochondria to donor-like levels [32].

In conclusion, the use of LVAD in the modification of the myocardial cell metabolism is inextricably linked to cardiac recovery. By restoring the transcriptional regulation of genes key to metabolism, increasing the activity of specific enzymes and proteins and restoring the amount of oxidative substrate for energy production, the supply of energy to the cell is increased to improve its functioning. Further, with the diversion of the glucose metabolism we have an increase in the production of important structural and functional elements necessary for the repair of cellular structures and the production of reducing molecules that protect against harmful exposure to oxidative stress. Finally, regarding mitochondria, which are the main source of energy, there is a restoration of gene regulation, an increase in its functionality and an improvement in both the density of the tumor and its normal distribution.

### 4.7. Genome

In addition to the modifications mentioned above regarding the genes that control various processes involved in myocardial recovery (metabolism, apoptosis, cell structural proteins, etc.), studies also demonstrate a more general change in the amount and ability of the cellular genome to replicate after LVAD placement. Specifically, in a study in which the changes of the genetic material in heart failure and after LVAD placement were compared to healthy donors, the following conclusions were reached: (1) After LVAD placement, a reduction in the size of the myocardial nuclei was observed through a reduction in their perimeter and area of. It is also noted that the differences in terms of perimeter and area also existed between cells before LVAD placement and control group cells. (2) After LVAD placement, there was an increase in the population of cardiomyocytes with more than 2 nuclei and, in particular, an increase in the phenomenon of diploidy with parallel reduction of this polyploidy. On the contrary, differences in the number of diploid cells were not observed in cells before LVAD placement and in a control group. (3) Reduction of the total amount of DNA in the cells that had been unloaded through the LVAD and at the same time their amount did not show a statistical difference with those of the control group, indicating the tendency of the former to return to the normal cellular state. (4) There was a positive correlation between the average cell DNA content and the number of centromeres.

Studies have shown an increase in the phenomenon of polyploidy in heart failure and especially in cells that have shown pathological hypertrophy. It is hypothesized that, in the given situation, we have an increase in cells entering the cell division cycle and transitioning from the G1/S checkpoint to the S phase with the help of cyclins. In this phase, the cells increase their size, the size of their organelles and the amount of their proteins and DNA replication takes place. This explains the increase in the amount of DNA and polyploidy seen in heart failure due to hypertrophy. After LVAD placement and the improvement of cardiac function, we see a reversal of the above phenomena. It is likely that, due to the reduction of stimuli for hypertrophy after the unloading of the cells, the interruption of the process of cell division of cardiomyocytes is lifted and even accelerated. This explains the increase in binucleate cells, which are cells obtained during the replication process, and also the decrease in the polyploidy phenomenon [33]. We can therefore consider from the above data the benefit of LVAD in the restoration of the genome of cardiomyocytes and in myocardial regeneration as it promotes normal cell division, which is a necessary element in this process, and the reduction of hypertrophic cells, thus promoting reverse remodeling.

## 5. Side Effects

From studies conducted it was observed that ejection fraction, calcium homeostasis, BNP levels and reverse remodeling, while showing improvement with short-term use of LVAD, the benefit was offset by long-term indwelling. At the same time, an increased occurrence of cardiac atrophy was observed with the prolonged use of the device, which was associated with cardiac dysfunction. The pathophysiological mechanism that plays a central role in this process is described below.

CaMKII (calcium-calmodulin-dependent protein kinase type II), when it detects abnormal calcium levels, is activated by binding CaM (calcified calmodulin) and autophosphorylates at the Thr 135 region, which allows it to remain activated independently of CaM. It then activates histone deacetylases, translocating them from the nucleus to the cytoplasm. These in turn activate MEF2 (cardiomyocyte transcription enhancer), which increases the expression of embryonic cardiac genes and stress response genes such as MSTN (myostatin growth factor regulator negative) which conduct anti-hypertrophic signaling, resulting in thinning of the ventricular wall as occurs in advanced heart failure.

Researchers, at the molecular level, also observed that: (1) Increasing the duration of LVAD use resulted in increased activation of cytoplasmic CaMKII (not nuclear) which was not the case with short LVAD use. (2) Increased LVAD indwelling results in an increase in phosphorylation of HDAC4 and nuclear MEF2 as opposed to cytoplasmic, probably due to increased translocation of the factor to the nucleus for the transcription of anti-hypertrophic genes. (3) Long-term use of LVAD increases the expression of Activin A (joint actions with MSTN), the ratio of phosphorylated SMAD 2/3 to SMAD 2/3 (in the short term it tended to decrease) and cardiac BMP1 (bone morphogenic protein) expression, where the latter two are involved in enhancing the action of the MSTN signaling pathway with BMP1 associated with a reduced degree of reverse ventricular remodeling. (4) Short-term LVAD use was associated with a reduction in apoptosis. (5) Comparing m changes of specific proteins in HF and after LVAD placement, a decrease in the ratio of phosphorylated ERK42 / ERK42 and phosphorylated ERK44/ERK44 (extracellular signal-regulated kinase) was observed, p 38 MAPK phosphorylation remained unchanged, AKT phosphorylation did not change statistically significantly, phosphorylation of the cell growth regulator mTOR (mechanistic target of rapamycin) remained elevated, stress marker CRF1 (corticotropin-releasing factor receptor) increased while CRF2 (which is involved in Akt and ERK activation) decreased. The above proteins have an important role in promoting cardiac muscle hypertrophy. Further, there was no change in their activation with regard to the duration of the LVAD placement. (6) Of the structural proteins, Actin and MPSI remained unchanged, while desmin, which is an indicator of promoting hypertrophy, decreased after the use of an LVAD device.

These data show a reduction in the activation of molecular pathways leading to cardiac atrophy, which is reversed by increased duration of LVAD placement. At the same time, the molecular pathways that promote muscle hypertrophy do not show many changes, regardless of the duration of abdominal unloading. We conclude that, in the initial period of LVAD operation, suppression of atrophic and activation of hypertrophic processes contributes to the restoration of cardiomyocyte structural elements and heart mass. On the other hand, in the prolonged duration of ventricular unloading, they activate the mechanisms promoting atrophy, resulting in the removal of the favorable effects of the LVAD and further cardiac dysfunction. It is possible that, due to the thinning of the wall, a reduction in the ejection fraction is caused, ultimately leading to congestive heart failure symptoms [34]. On the contrary, the study by Diakos et al. supports the non-induction of atrophic remodeling with the use of continuous flow LVAD [35]. Therefore, there is a need to design studies in the future to clarify the possibility of causing atrophy in mechanically supported hearts dependent on the type of device as well as considering the involvement of drugs that are considered to have a protective effect on this phenomenon such as Clenbuterol, which has been introduced in the Harefield protocol.

Additional studies point to potential right ventricular strain from prolonged LVAD use. This occurs as these systems increase right ventricular preload while also causing a leftward shift of the ventricular septum. In this way, a chronic burden on the right ventricle is created, where the maintenance of this functionality is vital both for delaying the progression of heart failure and for the patient’s potential for future transplantation. Another element is with reference to studies of increased activation of the sympathetic system in continuous flow LVADs. The most likely explanation is considered the fact of over-activation of baroreceptors due to the absence of pulsatility, resulting in increased sympathetic activation with all the adverse consequences this causes. Finally, the finding of increased molecular markers of inflammation such as CRP, IL-7, IL-8, TNF etc. during the stay of the LVAD leads to the conclusion of the increased inflammatory environment possibly as a foreign body type reaction [2] inducing possible fibrotic phenomena in the myocardium with a serious negative impact on the recovery of the organ.

## 6. Weaning from LVAD Studies—Patient Selection

For this reason, several studies have been conducted on the possibility of maintaining recovery after LVAD removal as well as on the characteristics of patients in whom recovery is promoted (Table 1). Listed below are the most important studies in the literature as well as characteristics of the patients who participated or recovered (depending on the paper).

From the above studies, we conclude that the characteristics: younger age, non-ischemic heart disease, shorter history of heart failure, shorter time of LVAD support, use of heart failure medication and reduced LV end diastolic diameter (LVEDD) are factors in favor of myocardial recovery in patients with an LVAD device. In addition to the phenotypic characteristics, the “reverse ramp protocol test” also plays a major role in predicting myocardial recovery. In this, the speed of the LVAD rotor is gradually reduced per minute to the point where the heart is not mechanically supported by the device without simultaneously increasing the risk of its clotting (final values HeartMate II 6000 rpm, HeartMate III 4000 rpm, HVAD 1800 rpm) [47]. Measurements are then taken with the now more common Harefield protocol for LVAD weaning. In the first stage, an ultrasound is performed before reducing the speed of the rotor to LVEDD < 60 mm, LV end systolic diameter (LVESD) < 50 mm, LVEF > 45% without changes after reducing the speed of the device; in the second stage a cardiorespiratory test is performed where PVO2 > 18 mL/kg/min and VE/VCO2 < 34; in the third stage right catheterization is performed where CI > 2.12 L/min and pulmonary capillary wedge pressure (PCWP) < 15 mmHg and finally; in the fourth stage, LVAD removal surgery is performed should the measurements in stage three apply when blocking the outlet cannula of the device [48]. In addition, published studies indicate several measures that could potentially predict myocardial recovery, such as pre-LVAD maximum LV Torsion [49], AP ventricular dilation time constant (tau) and LV dp/dt when measured with left cardiac catheterization, with the former being reduced and the latter increased in myocardial recovery [50] while also the former is a predictor of survival from hospitalization for heart failure and for reverse remodeling [51]. Finally, markers such as increased end-diastolic relative wall thickness (RWT) before LVAD removal, increased mean arterial pressure (MAP) during the 6 min walking test (6MWT) and increased S’ wave > 8 cm/s show increased correlation with recovery after LVAD weaning [52].

Data on the role of nuclear medicine techniques in the prediction of myocardial recovery after LVAD implant remain scarce. An interesting study, including 18 clinically stable patients supported with the second-generation axial flow LVAD (median duration of LVAD support 7 months), examined the effect of hemodynamic unloading on myocardial viability with the use of technetium-99m ((99m)Tc)-sestamibi single photon emission computed tomography (SPECT) imaging [53]. The researchers observed no significant change in viable myocardium, globally (median Δ 0.10%, IQR −1.7, 2.2, *p* = 0.80; 95%CI 0.95, 1.00) and regionally (median Δ 0.10%, IQR −1.5, 1.9, *p* = 0.88; 95%CI 0.93–1.00), between baseline and after an interval of 2 to 3 months, while patients were at reduced LVAD support. However, a recent study investigated whether long-term LVAD mechanical unloading results in metabolic activation of quiescent myocardial regions by examining myocardial 18F-fluorodeoxyglucose (FDG) uptake in 4 patients with end-stage HF after LVAD implantation [54]. It was concluded that all participants exhibited some degree of increase in FDG uptake in areas of previous metabolic inactivity at baseline, suggestive of possible myocardial regeneration. Therefore, further studies are needed to determine the effect of mechanical unloading on myocardial viability.

Therefore, although the international literature shows low rates of myocardial recovery in patients where LVAD placement has been performed, a great heterogeneity is observed in this work regarding the study population and its characteristics (age, heart disease, etc.) as well as the treatment and evaluation protocols of myocardial recovery internationally [55]. Elucidation of certain patient characteristics, as well as specific imaging and molecular markers such as those analyzed in the paper, could contribute to the creation of algorithms that will safely predict which patients will be suitable candidates for myocardial recovery. There also seems to be a need to adopt common international criteria for pharmaceutical treatment and evaluation. With the implementation of the above measures, it is likely that, in the future, the recovery rates will increase to a large percentage along with the developments presented through the minimally invasive techniques of removal of the systems that present significant advantages over the current ones.

## 7. Treatment of Arrhythmias and Use of CRT

In addition to studies on the recovery of the myocardium from direct damage to it, there is now also great interest in myocardial recovery in patients who have experienced heart failure as a result of a chronic burden of arrhythmias. The most characteristic is the NEw-Onset LBBB-Associated Idiopathic Nonischemic CardiomyopaTHy (NEOLITH) II substudy which highlighted the improvement of myocardial function through the improvement of the ejection fraction and the reduction of events in patients with non-ischemic cardiomyopathy caused by left bundle branch block (LBBB NICM) after synchronization therapy with CRT. The characteristics found in responders to CRT therapy are (1) absence of hypertension and (2) lower blood urea nitrogen (BUN) levels and reduced heart rate [56]. The literature also mentions the reduction of the mass and volume of the left ventricle [57] and the reduction of the functional insufficiency of the mitral valve (2) after the use of the above systems. The molecular mechanisms assumed to be involved are related to the improvement of the functioning of the Na^+^, K^+^, Ca^2+^ channels and, by extension, an improvement of the calcium cycle in the cell which will lead to a reduction in the duration of the depolarization potential resulting in the reversal of the desynchronization of ventricles. At the same time, the involvement of CRTs in the mechanisms of mitochondrial genome regulation, modulation of molecular signaling and inhibition of apoptotic signals has been speculated [58]. In addition, a specific study highlighted the improvement of cardiac function after reversal of any type of arrhythmia and its duration, with the exception of patients with an increased number of spontaneous ventricular contractions. Finally, in another study on the improvement of cardiac function and the achievement of reverse remodeling in pediatric tachycardia-induced cardiomyopathy (TIC) after treatment with pharmaceutical and interventional methods, an increased ejection fraction, younger age and increased heart rate were statistically significant indicators while small left ventricular dimensions were associated with reverse remodeling [59].

From the above data, we first observe that, with the above interventions in specific arrhythmological conditions, the phenomenon of myocardial depression is achieved. Further, especially for CRT, the data advocate for the increased possibility of myocardial recovery with the criteria discussed above. There is therefore a need to design and conduct new studies with patients with specific inclusion criteria to certify the achievement of myocardial recovery in specific populations with specific arrhythmic conditions.

## 8. Medical Treatment and Cardiac Rehabilitation

Several studies, both prospective and retrospective, have demonstrated the association between the use of neurohormonal inhibitors and reverse remodeling in patients with LVADs [42,43,60,61]. An interesting retrospective analysis of 12,144 LVAD patients from the Interagency Registry for Mechanically Assisted Circulatory Support (INTERMACS) revealed that those receiving any neurohormonal inhibitor at 6 months had a better survival rate at 4 years compared with patients not receiving neurohormonal inhibitor (56.0% vs. 43.9%) [62]. Interestingly, patients who were on neurohormonal inhibitors exhibited better functional capacity (assessed by 6MWT) and quality of life (assessed by Kansas City Cardiomyopathy Questionnaire score). On top of optimal guideline medical treatment, existing evidence shows that cardiac rehabilitation may benefit LVAD patients and facilitate myocardial recovery (i.e., metabolic changes in the failing myocardium and anabolic effects) [63,64]. A recent position Statement from the Heart Failure Association (HFA) of the European Society of Cardiology (ESC) highlights the importance of the patient’s screening to avoid complications as exercise training prescription should be individualized to meet the patient’s needs [64].

## 9. Conclusions

**Figure 4:**
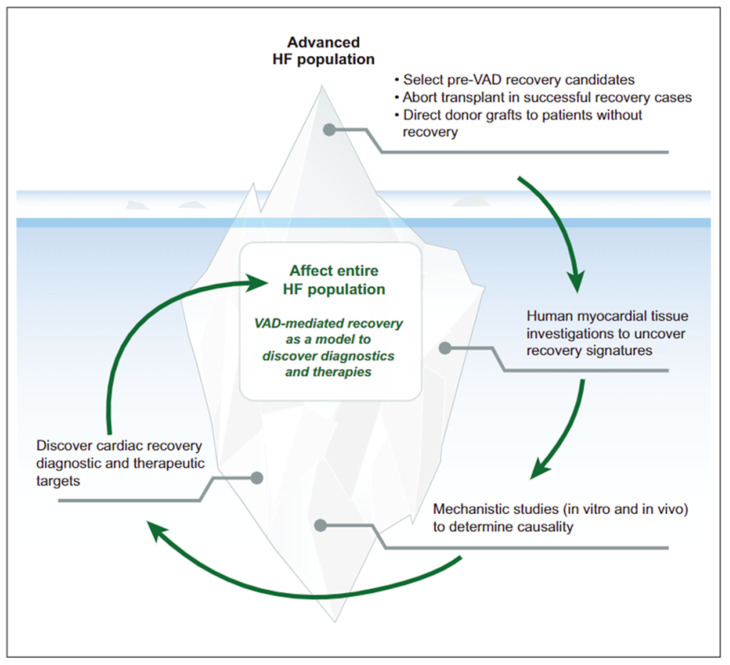
Extrapolating the lessons learned from patients with ventricular assist devices to the broader heart failure population. Reprinted with permission from Taleb I, et al., (2022), Copyright © 2022, Wolters Kluwer Health [65].

**Figure 5:**
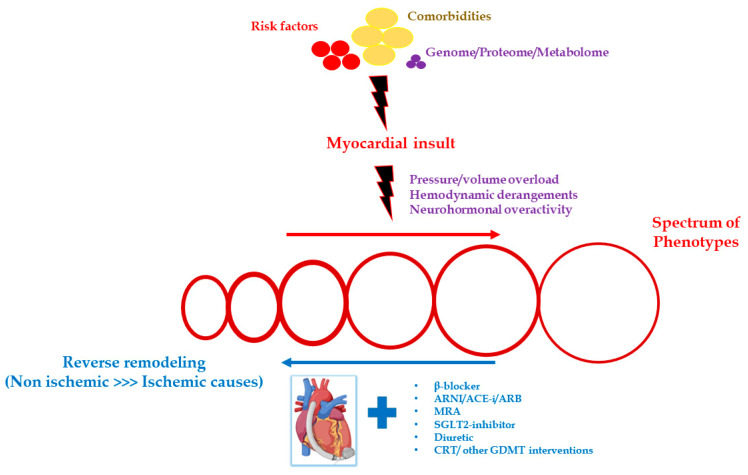
Heart failure (HF) is a spectrum of phenotypes. Each HF phenotype is the result of a patient-specific trajectory wherein the heart remodels towards concentric hypertrophy, eccentric hypertrophy or a combination of both. The way of entry and the subsequent path of the trajectory depend on the patient’s risk factors, comorbidities and disease modifiers (genome, proteome, metabolome). Both pharmaceutical treatment (i.e., β-blocker, ARNI/ACE-i/ARB, MRA, SGLT-2 inhibitor, diuretic) and device therapy (i.e., cardiac resynchronization therapy, left ventricular assist devices) may lead to reverse remodeling. Abbreviations: ARNI, angiotensin receptor-neprilysin inhibitor; ACE-I, angiotensin-converting enzyme inhibitor; ARB, angiotensin receptor blockers; MRA, mineralocorticoid receptor antagonists; SGLT2-inhibitor, sodium-glucose Cotransporter 2-nhibitor; CRT, cardiac resynchronization therapy; GDMT, guideline medical therapy.

From this work, specific conclusions are drawn about the feasibility of myocardial recovery. Initially, it is evident that it can exist as a phenomenon given that it has repeatedly described in many studies but also a number of involved pathophysiological mechanisms have been identified (Figure 4) [65]. The criteria for its definition are also set to avoid misinterpretations and wrong generalizations. The fact of the low and varying success rate of the interventions analyzed above, as well as the partial reversal of the pathophysiological mechanisms leading to heart failure, highlights that myocardial recovery manifests in a continuous spectrum rather than an all-or-none phenomenon. It directly depends on the type of damage to which the heart is subjected, the therapeutic intervention and the clinical characteristics of patients. Thus, there is a need to design studies with disease- and intervention-specific patient inclusion criteria, consistent medication protocols and monitoring protocols to determine true myocardial dimensions for recovery. Myocardial recovery should be an intensive field of study in the coming years [61,65]. This is evident from the tendency of now official organizations to recategorize these patients, for example, the American Heart Association (AHA), which created the HFimpEF classification for patients, studied as a separate group those who, after therapeutic interventions, improved their cardiac function (Figure 5). With the development of technology and the expansion of the use of neural networks and artificial intelligence, it is hoped that individual algorithms will improve the prediction of the potential of patients for myocardial recovery. This will benefit them, by offering the most and highest quality years of survival, but also reduce the number of candidate patients on heart transplant waiting lists.

## Figures and Tables

**Table 1:** Myocardial recovery studies in patients with LVADs.

Study	Conducted	Characteristics	Outcome
Thoratec registry [36]	1990–1999	Characteristics: 22 patients with non-ischemic heart failure (12 myocarditis, 4 sarcoid cardiomyopathy, 1 viral, 2 idiopathic), 12 female, mean age 32 years, duration of support 57 days, use of Thoratec device (pneumatic LVAD)	Benefit of LVAD in acute myocarditis
Berlin Group [37,38]	1995–2004 and then until 2008	32 patients from a total of 131 with dilated cardiomyopathy. Eligible: 30 men, 4.5 months average LVAD support Follow-up study 2008. Of 188 patients with idiopathic dilated cardiomyopathy in 30 LVAD removal. Characteris-tics: LVEF 30–44% and LVEDD 56–60 mm at device removal, use of LVAD, BiVAD, RVAD systems. Results: probability of survival 5 and 10 years after LVAD weaning when at 1 year there was no recurrence of heart failure 84% and 61%, respectively	Of the 32, 4 died 2 for cardiac reasons, 2 for non-cardiac reasons, remaining survival >3 years. Overall study conclusion: LVEDD > 55, LVEF < 45% before LVAD removal and HF duration ≥ 5 years are poor prognostic factors. Patients having 2 of the 3 factors have a reduced chance of recovery Patients with a shorter history of heart failure, were younger and required less time on LVAD support have an increased likelihood of recovery
University of Pittsburgh study [39]	1996–2003	Of 154 subjects with LVAD, removal was considered possible in 10. Characteristics: 2 ischemic and 8 non-ischemic etiology (4 peripartum cardiomyopathy, 3 myocarditis, 1 idiopathic), 120 days mean support time, mean age 30 years, 88% women	LVAD support offers better outcomes in patients with gestational cardiomyopathy and myocarditis
IMAC2 (Intervention in Myocarditis and Acute Cardiomyopathy) cohort [40]	2002–2008	14 patients with acute myocarditis had LVAD inserted, 8 were candidates for recovery. Characteristics: mean age 30 years, 38% male, 10 with pulsatile LVAD 4 with continuous flow, implantation 1 month after symptoms, in recoveries increased inflammation, little fibrosis and reduced LVEDD while in non-recoveries vice versa	Increased likelihood of recovery in recent-onset cardiomyopathy, biopsy and LVEDD guide potential recovery
MCS UNOS (United Network Organ Sharing) registry [41]	2005–2013	Out of 686 patients, LVAD removal was performed due to recovery in 34. Characteristics of persons with recovery: average age 40 years, women 41%, 33 HeartMate II and 1 Heartware device and average duration of support 382 days while in 66% of them recovery maintained after 1 year	Patients who experienced recovery were younger, female, had non-ischemic cardiomyopathy, had a lower BMI, had not had a prior ICD implanted and had a lower serum creatinine
Montefiore Medical Center, Albert Einstein College of Medicine, Bronx, New York [42]	2006	21 patients (8 with coronary artery disease, 13 with idiopathic dilated cardiomyopathy) of 34 initially placed with an LVAD were given neurohormonal blockade and attempted weaning. Characteristics of selected patients: mean age 48 years, disease duration before LVAD up to 821 days, 20 HeartMate II and 1 VentrAssist device. Results: 16 subjects developed reverse remodeling and after device 3 downshift control weaned	The use of neurohormonal blockade aids in reverse remodeling while reduced pre-LVAD disease duration, less fibrosis, less hypertrophy and increased LVAD turns increase the likelihood of weaning
Harefield Study [38,43]	2006–2009	15 patients received a pulsatile flow LVAD and pharmacologic protocol for heart failure + clebuterol with 11 having the device removed. Characteristics of patients who recovered: LVEF at withdrawal 65 % mean, LVEDD 56 mm, 321 days of support. Final results: freedom from heart failure deregulation at 1 and 4 years 100% and 89%, respectively 20 patients with nonischemic cardiomyopathy were implanted with a HeartMate II continuous flow LVAD device in combination with neurohormonal blockade and clebuterol. Patient characteristics before LVAD placement: age 16–58, LVEDD 57–91 mm, LVEF 7–34%, PCWP 31 mmHg, supported by inotropes, 16 were male, mean age results: 10 patients experienced 1–3 years of recovery with 66% having experienced heart failure prior to LVAD implantation up to 6 months prior. Further, before device removal at low flow they had a mean LVEF of 70%, LVEDD of 48 mm, PCWP of 6 mmHg	Medication and pulsatile flow LVADs promote recovery. The use of continuous flow LVAD in combination with medication can promote myocardial recovery
INTERMACS (Interagency Registry for Mechanically Assisted Circulatory Support) Registry [44]	2006–2015	Of 13,454 LVAD patients 163 had recovery capable of weaning from the device and 8805 had partial recovery. Characteristics of patients with LVAD removal: Mean age 45 years, 38.7% female, 85.9% non-ischemic cardiomyopathy, 95.7% axial flow LVAD, mean duration of support 16 months	Younger patients (<50), of non-ischemic etiology, with LVEDD < 6.5 cm, PASP < 50 mmHg, time to heart failure diagnosis < 2 years, with axial flow LVAD systems, without optimal medication, had an increased chance of recovery treatment and with diagnoses of myocarditis, cardiomyopathy of pregnancy and cardiomyopathy from the use of andriamycin. Finally, patients in whom a pulsatile flow LVAD was placed had a greater chance of recovery compared to those in continuous flow LVADs
Utah Transplantation Affiliated Hospitals Cardiac Transplant Program [45]	2008–2014	154 consecutive patients with documented chronic and dilated cardiomyopathy (ischemic cardiomyopathy/ICM, n = 61; non-ischemic cardiomyopathy/NICM, n = 93) requiring durable support with continuous-flow LVAD were prospectively evaluated with serial echocardiograms and right heart catheterizations Among patients supported with LVAD for at least 6 months, 5% of subjects with ICM and 21% of subjects with NICM achieved left ventricular ejection fraction ≥40% (*p* = 0.034). LV end-diastolic and end-systolic volumes and diastolic function were significantly and similarly improved in patients with ICM and NICM	LVAD-associated unloading for 6 months resulted in a substantial improvement in myocardial structure, and systolic and diastolic function in 1 in 20 ICM and 1 in 5 NICM patients
RESTAGE-HF (REmission from Stage D Heart Failure) Study [46]	2012–2015	Of the 40 patients selected, 36 were able to implement the protocol and of these 16 eventually had the LVAD removed. Characteristics of selected patients: mean age 35 years, 68% male, with non-ischemic heart disease of less than 5 years’ duration, 95% with fibrotic support, 17.5% with temporary mechanical support, with mean LVEF values of 15%, and LVAD device the HeartMate II. Final results: of the 36 patients, 16 underwent LVAD removal where they survived 12 months after removal and 14 of these tended to reach 3 years. Further, only serum creatinine was statistically significant in predicting recovery	The use of specific drugs as well as the monitoring of patients with a specific protocol increases the likelihood and duration of recovery after removal of LVAD systems [46]

## Data Availability

Not applicable.
